# Normal Ovariotomy

**Published:** 1872-09

**Authors:** 


Normal Ovariotomy.—The article upon this subject, in which is reported an operation for the extirpation of the normal ovaries, by Dr. Robert Battey, of Rome, Georgia, will, of course, attract attention. The Doctor is a bold and eminently skillful surgeon; and whether the propriety of the operation is clearly demonstrated or not, he certainly presents a strong case in its favor—certainly of sufficient weight to relieve him of any charge of rash or reckless surgery. He writes us that he is prepared to be impaled upon many a lance of criticism, but believes that the operation will sooner or later be admitted, by the mass of right-thinking men in the profession, to be entirely justifiable.

